# Nanotechnology-based approaches for targeting and delivery of drugs via Hexakis (m-PE) macrocycles

**DOI:** 10.1038/s41598-021-87011-6

**Published:** 2021-04-15

**Authors:** Samaneh Pasban, Heidar Raissi

**Affiliations:** Department of Chemistry, University of Birjand, Birjand, Iran

**Keywords:** Biochemistry, Cancer, Chemistry, Nanoscience and technology

## Abstract

Hexakis (m-phenylene ethynylene) (m-PE) macrocycles, with aromatic backbones and multiple hydrogen-bonding side chains, had a very high propensity to self-assemble via H-bond and π–π stacking interactions to form nanotubular structures with defined inner pores. Such stacking of rigid macrocycles is leading to novel applications that enable the researchers to explored mass transport in the sub-nanometer scale. Herein, we performed density functional theory (DFT) calculations to examine the drug delivery performance of the hexakis dimer as a novel carrier for doxorubicin (DOX) agent in the chloroform and water solvents. Based on the DFT results, it is found that the adsorption of DOX on the carrier surface is typically physisorption with the adsorption strength values of − 115.14 and − 83.37 kJ/mol in outside and inside complexes, respectively, and so that the essence of the drug remains intact. The negative values of the binding energies for all complexes indicate the stability of the drug molecule inside and outside the carrier's cavities. The energy decomposition analysis (EDA) has also been performed and shown that the dispersion interaction has an essential role in stabilizing the drug-hexakis dimer complexes. To further explore the electronic properties of dox, the partial density of states (PDOS and TDOS) are calculated. The atom in molecules (AIM) and Becke surface (BS) methods are also analyzed to provide an inside view of the nature and strength of the H-bonding interactions in complexes. The obtained results indicate that in all studied complexes, H-bond formation is the driving force in the stabilization of these structures, and also chloroform solvent is more favorable than the water solution. Overall, our findings offer insightful information on the efficient utilization of hexakis dimer as drug delivery systems to deliver anti-cancer drugs.

## Introduction

Nano-carriers based delivery platforms have developed as a promising candidate for cancer treatment. However, their quality problems (i.e., the inability to tune the pore diameters and difficult production), carrier-related toxicity, and poor drug loading capacity issues have restricted their clinical utilization^1–3^. Nevertheless, the research interest has shifted to focus on the design and fabrication of novel drug-delivery vehicles^4–10^. Among the different ring-shaped macrocycles, those with non-deformable cavities and rigid backbones are of particular interest. Because the stacking of such macrocycles creates structures with strictly defined inner and outer diameters, along with the internal pores^11,12^. One kind of these ring-like structures is based on oligo-(phenylene ethynylene) rigid backbones that have also been widely studied^13^. These oligomers are molecular-scale (< 2 nm) porous structures, called nanopores, with fixed sizes and interesting mass-transporting properties, which are commonly known as useful biocompatible materials. Until now, several different classes of planar and rigid macrocycles have been reported that provide attractive building blocks for the formation of nanotube assemblies containing internal pores with defined diameters^14^. Despite carbon nanotubes (CNT) and inorganic porous materials, the rigid building blocks of macrocyclic are endowed with the ability to be functionalized at defined locations inside the tube^15^. One class of these structures is Hexakis (m-PE) macrocycles that are a series of rigid macromolecular structures consisting of m-phenylene–ethynylene units. The (m-PE) macrocycles exhibit completely different self-assembling behaviors that are influenced by multiple factors. For example, Moore et al.^16^ revealed that the Hexakis macrocycles are able to form a self-assembly tubular structure in low-polar solvents due to π-stacking and intermolecular H-bonding interactions. As well as, the results from our previous study^17^ via molecular dynamics (MD) simulations indicated that Hexakis macrocycles undergo a self-assembly process forming a nanotubular structure that can be the biocompatible potential sensor for drug delivery applications. Doxorubicin (DOX), an anthracycline anti-cancer antibiotic, is among the most potent antitumor agents used for the treatment of a variety of malignancies, including breast, prostate, brain, cervix, and lung cancers^18^. Despite extensive clinical utilization, the DOX application is severely limited because of the risk of severe cardiotoxicity^19^. Besides, this anti-cancer drug also affects other organs like the kidney, liver, and brain^20,21^. Therefore, to improve the therapeutic effect and minimize the side effects, it is necessary to develop new therapeutic strategies for the selective delivery of DOX to tumors. Many research groups have tried to design a new generation of intelligent drug delivery systems to efficiently carry doxorubicin to destroy cancer cells^22–25^. These days computational chemistry is considered as one of the most important branches of physics and is playing an increasingly important role in various fields such as biological, chemical, and material sciences. In addition, computational approaches are an alternative method for identifying the mechanism of the adsorption process and exploring highly efficient adsorbents, which can determine the adsorption capacity of materials at special sites. On the other hand, advances in computational chemistry have the potential to provide newer and faster screening methods. Computational model systems as the field of host–guest chemistry are tractable and yet informative for biomolecular recognition. In fact, the modeling predictions conducted as part of the host–guest affinity challenge, are generally in good quantitative agreement with the experimental work^26^. Thus, theoretical prediction can assist the experimental chemist or it can challenge the experimental. For example, a host–guest complex between MOF as host and doxorubicin drug as a guest is investigated using an experimental and theoretical study to explore MOF potential application for DOX delivery by Junior et al.^27^. The experimental studies are performed by XRPD, FTIR, and UV–Vis spectroscopy to investigated the DOX loading, release profile, and cytotoxicity in vitro. Besides, theoretical models are used to explain and understand the interactions between DOX drugs and MOF. The obtained results demonstrated that this system is effective in cancer treatment and minimizing its side effects, which is attributed to interesting stopper effects in guest–host interactions. Also, results showed that the theoretical studies are in good agreement with the experimental results. Since theoretical computer science provides a way to predict response to specific therapy and also to offer novel drug options, the present study density functional theory (DFT) calculations is carried out to provide an efficient approach for using hexakis dimer as a novel nanocarrier for DOX drug loading. For this purpose, the hexakis dimer's ability for doxorubicin delivery is examined by using DFT studies to determine the structural parameters, binding energy, and electronic properties of DOX/hexakis dimer complexes in chloroform and water solvents. By studying the hexakis dimer, we intend to answer the question: Do hexakis dimer, similar to tubular stacks, serve as a host molecule and form an inclusion complex with the guest drug?

In fact, the obtained results from this work highlight the utility of computational models in the host–guest binding affinity challenge as well.

## Computational details

In the present work, the structures of the monomer/dimer of the hexakis and dimer/guest inclusion complexes are optimized using the DFT (M06-2X) and DFT-D3 (M06-2X-D3) functionals^28,29^ by employing the 6-31G** basis set^30,31^ in the chloroform and water solvents. The initial geometry of the hexakis carrier is taken from the X-ray data, reported by Zhong and et al.^14^. In our work, the initial structures of hexakis dimer are built by using Gauss View software^32^, and the size of the side chain is derived from the experimental work, which is proposed by Zhong and co-workers. Therefore, in our model, the distances between aromatic stacking and H-bonded side-chain are 3.46 Å and 4.9 Å, respectively, where two monomers of the hexakis are placed at an angle of 20° respect together. Besides, the van der Waals diameter of this monomer is about 6.4 Å. Schematic representation of the hexakis monomer and guest molecule is illustrated in Fig. S1. It is worth noting that for choosing the most stable configuration of the drug-carrier complexes, the conformational search is carried out by using the Spartan software package^33^. To explore the hexakis dimer's ability in response to organic an aqueous solvent, the polarizable continuum model (PCM) is applied^34^. All the above calculations are performed by employing the Gaussian 03 package^35^. The strength of the adsorption is determined by computing the binding energy (∆E_ads_) that can be obtained from the following relation:

1 $$\Delta {\text{E}}_{\text{ads}}={\text{E}}_{\text{hexakis dimer}+\text{drug}}-\left({\text{E}}_{\text{hexakis dimer}}+{\text{E}}_{\text{drug}}\right)$$

Since the DFT-D method can well describe the geometries and energies of the non-covalent interactions, we employ the density functional theory including dispersion-correction (DFT-D3) as suggested by Grimme et al. The DFT-D3 energy (E_DFT−D_) is estimated by applying an empirical correction energy E_disp_ to the DFT energy (E_DFT_) to account for the effect of dispersion interactions, as follow:

2 $${\text{E}}_{\text{DFT}-\text{D}}={\text{E}}_{\text{DFT}}+{\text{E}}_{\text{disp}}$$

The analysis of bonding interaction between the DOX and hexakis dimer carrier has been performed using the energy decomposition analysis (EDA) via the Amsterdam Density Functional theory (ADF) package^36^ at the B3LYP-D/DZP level of theory. In the studied systems, the energy gaps (Eg) between HOMO (highest occupied molecular orbital) and LUMO (lowest unoccupied molecular orbital) are calculated to obtain the quantum chemical parameters such as the numerical values of the electronic chemical potential (μ), chemical hardness (η), and global electrophilicity index (ω) as shown in Eqs. (3–6)^37–39^:

3 $$ \upmu = \left( {{\text{E}}_{{{\text{HOMO}}}} + {\text{E}}_{{{\text{LUMO}}}} } \right)/{2} $$

4 $$ \upchi = - \upmu = - \left( {{\text{E}}_{{{\text{HOMO}}}} + {\text{E}}_{{{\text{LUMO}}}} } \right)/{2} $$

5 $$ \upeta = \left( {{\text{E}}_{{{\text{LUMO}}}} - {\text{E}}_{{{\text{HOMO}}}} } \right)/{2} $$

6 $$ \omega = \upmu^{{2}} /{2}\upeta $$

The total density of states (TDOS) and partial density of states (PDOS) of the DOX-hexakis dimer are calculated by using the MultiWFN 3.8 program^40^. Topology analysis, i.e., atoms in molecules (AIM)^41^ and Becke surface (BS)^42^ methods are carried out using the MultiWFN 3.8 program to confirm the existence, evaluation of hydrogen bonding (HB). To assess the type of interactions between the DOX and the hexakis dimer, the non-covalent interaction (NCI) calculation is carried out by the NCIPLOT code^43^. In addition, ^1^H NMR chemical shifts are calculated at the M06-2X/6-31G** level by using the gauge-independent atomic orbital (GIAO) approach^44^ and are given in ppm (δ) relative to tetramethylsilane (TMS) as an internal standard.

## Result and dissection

### Geometry optimization and adsorption energies

In this study, the hexakis dimer is investigated as a novel nano-carrier to predict its ability the deliver DOX to the tumor cells. The monomer and dimer structures of hexakis are optimized using the DFT method to investigate the impact of polar and non-polar solvents on the dimerization process of these structures. It should be noted that the stability between the hexakis (m-PE) rings is determined by the intermolecular H-bond interaction between the NH and CO (NH⋯O=C) groups. Nevertheless, the molecular nature of the solvents can affect the strength of the H-bond interaction between the hexakis (m-PE) rings and also the dimerization process. For this purpose, the effect of water (polar) and chloroform (non-polar) is studied. Moreover, all structures of the drug molecule, hexakis dimer, and DOX/hexakis dimer complexes are investigated in chloroform and water solvents to study the drug-carrier interacting system. Since the effectiveness of a drug is directly dependent on its molecular chemical structure, examine the structure parameters of the DOX drug is important after the adsorption inside and outside the carrier's cavities. The calculated bond lengths for the DOX molecule are reported in Table S3. According to the obtained results in this Table, the C–C and C–H bond lengths of DOX lie in the range 1.35–1.49 Å and 1.00–1.13 Å respectively. Also, the C-O bond distances are in the range 1.20–1.44 Å and the O–H bond distance is 1.05 Å. These results agree well with the experimental results (X-ray crystallographic data) reported by Courseille et al.^45^ In addition, the C–C–C bond angle values lie 120.1° and the C–O–C bond angle values lie 119.3°. As can be seen from the Table S4, in relation to the experimental data, our results reveal a good standard deviation (SD), which is another reason for good correlation between the experimental results and theoretical DFT calculated with 6–31 g** basis set. Consequently, we do not see any significant changes in the bond lengths of the DOX molecule after adsorption on the hexakis carrier. These observations confirm that the chemical structure of the DOX molecule is protected from degradation and cellular uptake is improved after complexation with hexaxis dimer. After the investigation of the dimerization process, it is found that the internal pores of hexakis dimer are well-preserved during the optimization. For both solvents, the calculated inner and outer diameters remained at 10.78 Å and 22.9 Å, respectively. It is worth noting that the (m-PE) macrocycles have a rigid backbone and do not allow free rotation about the axis of peripheral phenyl rings, thus making nondeformable cavities and a planar ring system. Our results is in good agreement with experimental data by Moore et al.^16^. Table S1 contains the optimized geometry parameters of the hexakis monomer and its dimer in chloroform and water solvents. The calculated N–H and C=O bond lengths of the monomer are (1.32 and 1.33) and (1.00 and 1.01) Å, while the corresponding lengths of its dimer are (1.33 and 1.34) and (1.01 and 1.02) Å in the chloroform and water, respectively. This finding is confirmed by the experimental values in ref^46,47^. Moreover, the calculated N⋯O distances in the two-neighboring macrocycle are approximately equal to 2.94 Å in both solvents, which these values are close to the distance between these atoms in the X-ray crystal structure (2.93 Å). Based on the obtained results, the N–H⋯O hydrogen bonds (HBs) between two plates of hexakis are perfectly formed, and also there is a strong π–π stacking interaction with a distance of 3.46 Å. The rigidity of this macrocyclic backbone gains its origin from the inter-planar attractions, mostly in form of π–π stacking interactions. The equilibrium HBs distances between carbonyl groups at the backbones with amide side chains of the adjacent is in the range of 1.97–1.98 Å and 2.00–2.02 Å in chloroform and water solvents, respectively. In the case of the N–H⋯O bond, this is alongside the lengthening of the N–H bond and shortening of N⋯O distance. It is well known that the shorter H⋯O distance can be devoted to the greater strength of the hydrogen bond and stronger HB energies (E*_HB_) in DOX/hexakis dimer complexes. Our results show that the shortest O⋯H and O⋯N distances and the strongest HB energies have been obtained for hexakis dimer in chloroform solvent, which are nearly equal to the previous theoretical data^48^. According to the above results, the dimerization is more favorable in the chloroform than water solvent. Therefore, it can be concluded that the hexakis dimer is more stable in chloroform, which is in agreement with our previous MD simulation^17^. In this study, two different positions are considered for the interaction of the DOX molecule with the hexakis dimer nanoporous. In one case, the DOX molecule is placed inside the hexakis dimer pores, and in the second case, the DOX molecule is placed outside the hexakis dimer near the entrance of the pores. It is worth noting that to study the absorption at the outside of the hexakis dimer, a distance of 2.5 Å between the carrier and the nearest atom of the DOX molecule is considered before optimizing.

**Figure 1:**
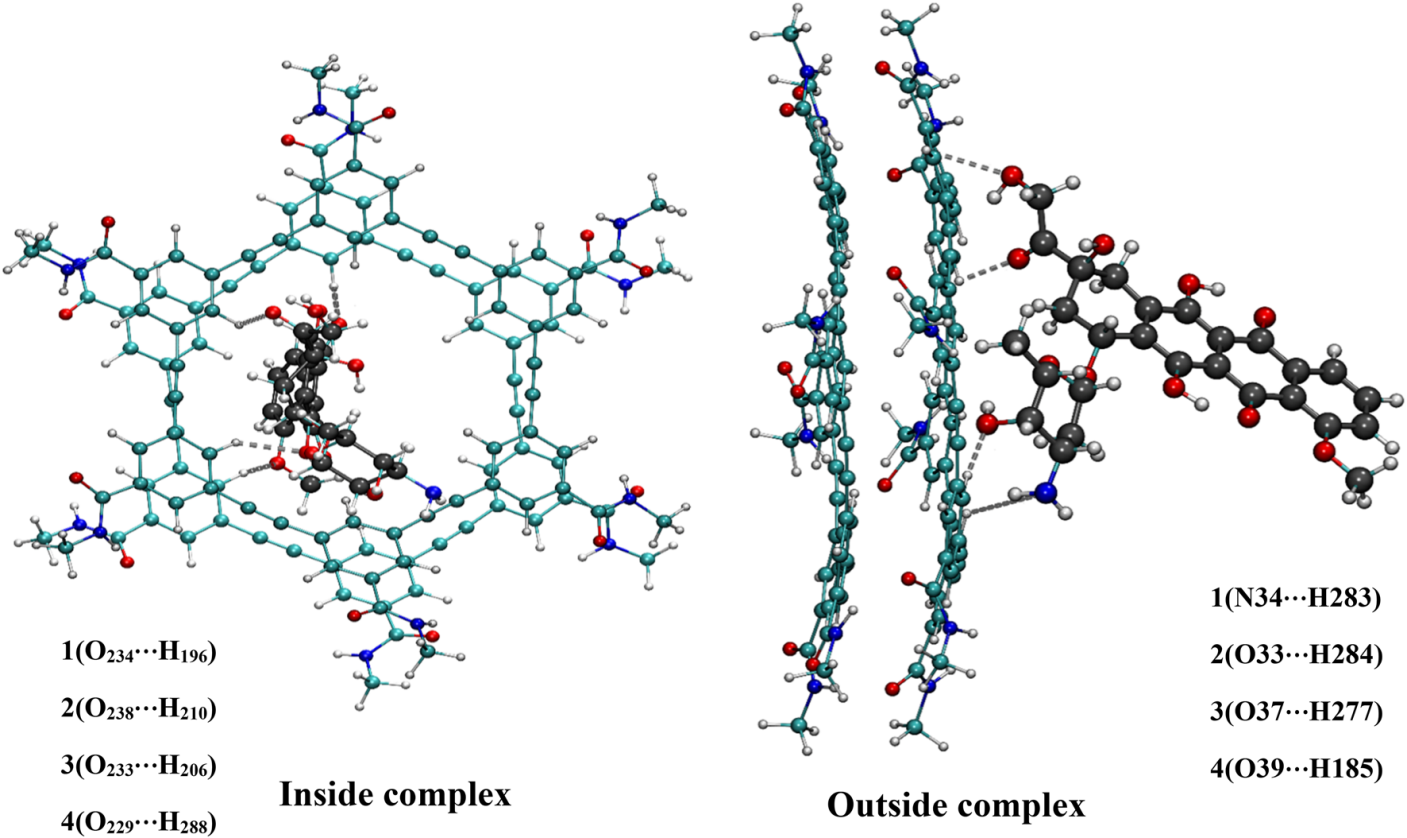
The optimized structures of inside and outside complexes along with the interatomic interactions numbering scheme.

In addition, the structural parameters for all complexes are listed in Table S2. Due to the complex formation between the DOX molecule and hexakis dimer nanoporous, the drug molecule is able to participate in various types of intermolecular hydrogen bonds such as conventional (N–H⋯O and N–H⋯N) and non-conventional (C–H⋯N and C–H⋯O) interaction (see Fig. 1.). As it is evident from Fig. 1, the drug molecule in inside complexes only involves non-conventional intermolecular HB with the carrier. In contrast, both non-conventional and conventional intermolecular HB are observed between DOX and carrier in the outside complexes. As can be seen in Table S2, the conventional HBs are significantly shorter and consequently stronger than that non-conventional ones in the studied complexes. Therefore, it should be noted that the hydrogen bonds could be an essential driving force for stabilizing the DOX/hexakis dimer in outside complexes. To investigate the strength of interaction of DOX molecule with the hexakis dimer nanostructure, the binding energy values are computed for the studied complexes with different functionals, regular and dispersion-corrected, and the results tabulated in Table 1.

**Table 1:** The adsorption energies (E_ads_) at the M06-2 × and M06-2x-D3 levels in chloroform and water solvents, the solvent energies (E_sol_) of the drug delivery systems.

Model	E_ads_ in chloroform	E_ads_ in water	E_DFT-D_ in chloroform	E_DFT-D_ in water	E_sol_
Inside complexes	− 83.37	− 72.12	− 131.78	− 123.70	− 35.9
Outside complexes	− 115.01	− 110.13	− 133.88	− 128.85	− 43.2

Unites of energies are in kJ/ mol.

According to the obtained results, the DOX-hexakis dimer complexes have considerable stability in different solvents. Data on this table exhibit that the binding energy values of the DOX-hexakis dimer complexes are more when the drug molecule locate outside the nanoporous microcycles. These results indicate that the more negative binding energy values result from the stronger intermolecular hydrogen bonds between the active sites of the DOX and the hexakis dimer nanopores in outside complexes. In addition, we noticed that the DOX molecule shows better interaction with the carrier in the chloroform solvent, as evidenced by the very high binding energies. This fact can be attributed to the polarity of chloroform solvent that significantly increases the stability of complexes compared to water. According to the results of Table 1, the utilization of the DFT-D approach showed that the binding energies of all the complexes become more negative. Indeed, the inclusion of Van der Waal corrections (DFT-D3) resulted in considerable improvement in the DFT calculation. The solubility and stability of the DOX-hexakis dimer complexes are assessed via the solvation energy (E_sol_), which is computed by the following equation:

7 $${\text{E}}_{\text{sol}}={\text{E}}_{\text{solvent}}-{\text{E}}_{\text{gas}}$$

where E_solvent_ and Egas are the total energies of the complexes in water solution and the gas phase, respectively. The solvation energies reported in Table 1 show that the solvation is a spontaneous process. The results show that the adsorption of DOX molecule at the outside and inside of the cavities is a physisorption process. Authors previously applied theoretical methods to investigate the sensitivity of CNTs as smart drug delivery systems^49^. Compared to our previous work on CNT nanotube, the obtained results indicate that hexakis macrocycles have much better performance for the load and delivery of anti-cancer agents. Moreover, both our former and current work emphasize on the importance of solvent effects in the process of complex formation.

### Energy decomposition analysis

The energy decomposition analysis (EDA) is an effective procedure for a quantitative interpretation of chemical bonds' interactions between molecules^50^. The EDA partition the total interaction energy into the most relevant terms such as dispersion energy (ΔE_dis_), electrostatic energy (ΔE_elect_), orbital (covalent) energy (ΔE_orb_), and repulsive exchange (Pauli) energy (ΔE_Pauli_). In this work, EDA analysis is considered to describe further the intermolecular interactions between the drug and nanocarrier in DOX-hexakis dimer complexes. For performing EDA analysis, the hexakis dimer is generally considered as one fragment, and the drug molecule as the other fragment, and the results are presented in Table 2.

**Table 2:** Energy (kJ/mol) decomposition analysis of DOX-hexakis dimer complexes with B3LYP-D/DZP level of theory.

Model	ΔE_elect_	ΔE_orb_	ΔE_dis_	ΔE_Pauli_	ΔE_int_
Inside complexes	− 60.02	− 40.64	− 76.23	95.09	− 81.98
	− 55.90*	− 36.40*	− 72.15*	92.16*	− 72.29*
Outside complexes	− 63.68	− 42.18	− 79.21	97.42	− 87.66
	− 61.92*	− 39.74*	− 75.68*	93.96*	− 83.38*

*In water solvent.

As it is seen from the results in Table 2, the dispersion term is the dominant interaction in the stabilization of all complexes. The ∆E_dis_ for outside complexes is higher than the inside complexes, which this result conforms with the binding energies. In addition to the ∆E_dis_ energy, the electrostatic term (− 63.68 kJ mol^−1^) also has a considerable contribution to stabilizing these complexes. Our findings are in remarkable agreement with previous studies^51,52^, indicating that the electrostatic and dispersion interactions are very likely the principal sources of the binding energy for the neutral hydrogen-bonded complexes. It worth mentioning that the contribution of the electrostatic term is much higher for outside complexes, compared to the inside complexes. This is mainly due to the electrostatic interaction between the –NH2 group of DOX and the oxygen atom of C=O in hexakis dimer, which this bond does not exist in inside complexes. In general, the results demonstrate that the ∆E_dis_ is not the only driving force for adsorption of the DOX drug onto the nano-carrier, but also the ∆_Eelect_ and ∆E_orb_ are playing significant roles in promoting stability. On the other hand, the orbital term can provide a solid guess of covalent energy's contribution to the attractive interactions. This finding is also in agreement with the QTAIM results, showing that the interaction of DOX with hexakis dimer is non-covalent in nature. Besides, the quantitative EDA analysis confirms that all terms become more stabilizing effect when the drug molecule is considered in chloroform solvent. Outcomes of EDA indicate that the total bonding energy is negative for all complexes. This result was in good agreement with the binding energy.

### AIM and BS analysis for the strength of H bonding

Further studies are required to better understand the strength and nature of the hydrogen bonding interactions in DOX-hexakis dimer complexes. Therefore, the AIM theory and Becke surface method are carried out in chloroform and water solvents. AIM analysis is a suitable approach in quantum mechanics to consider different types of interactions. The main ingredient of AIM theory is topology analysis of electron density. It is known that the ρ(r) and ∇^2^ρ(r) at the bond critical point (BCP) are correlated with the binding energy strength and shown the bond strength and bond characteristics. The hydrogen bond energy could be estimated using the Espinosa method based on the following equation:

8 $$ E_{{HB}}^{*}  = {\raise0.7ex\hbox{$1$} \!\mathord{\left/ {\vphantom {1 2}}\right.\kern-\nulldelimiterspace} \!\lower0.7ex\hbox{$2$}}V_{{(rCP)}}  $$

**Figure 2:**
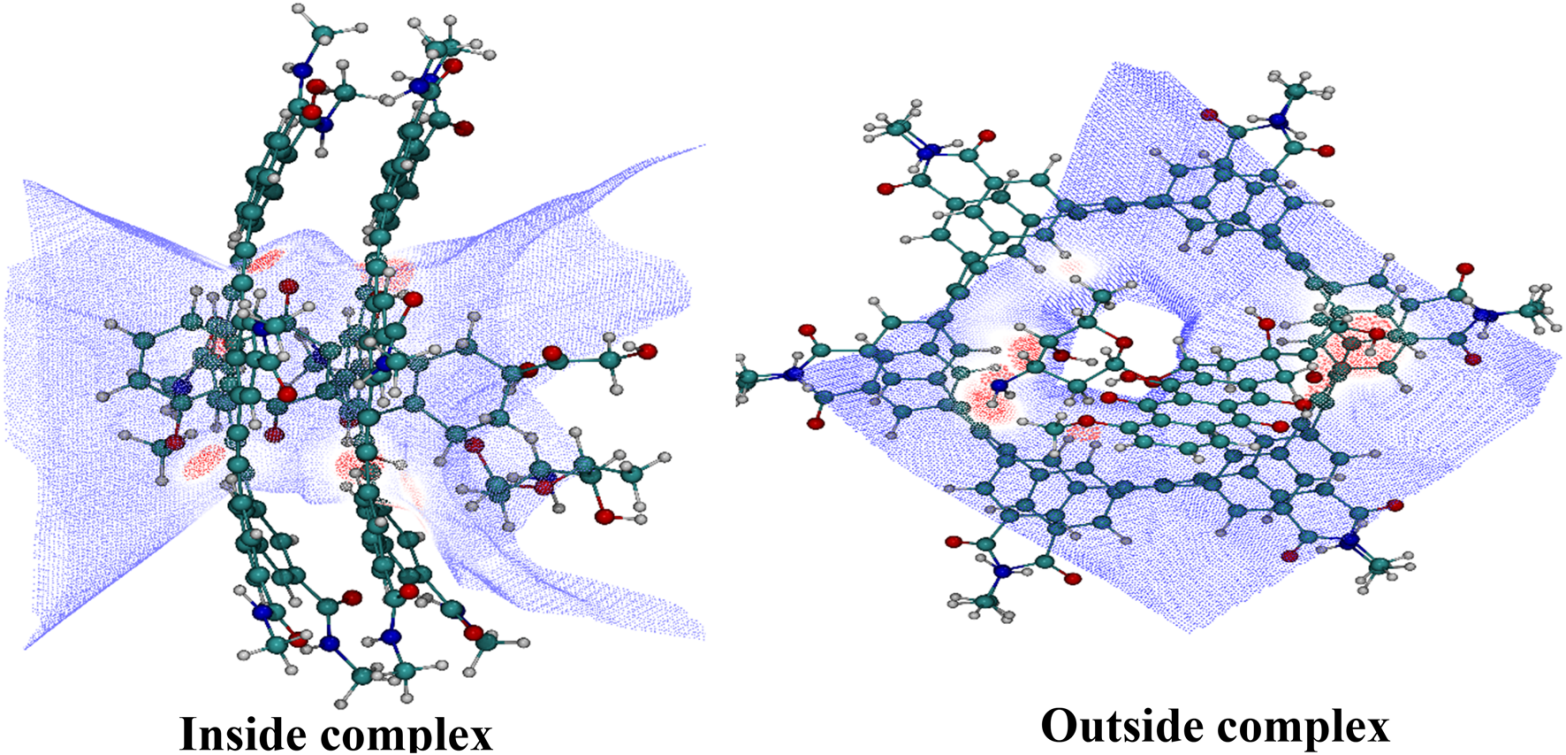
The colored Becke surface map of DOX drug in DOX-hexakis dimer complexes. Red zones in the maximized frames indicate regions of high electron density.

AIM representations of studied complexes, including bond critical point (BCP) and the bond paths, are presented in Fig. S2. The calculated values of total electronic density ρ(r), Laplacian electronic density ▽2ρ(r), and also energetic AIM parameters (kinetic energy density (G), the total energy density (H), potential energy density (V)) for considered complexes are presented in Table S3. According to this Table, the range of ρ(r) and ∇^2^ρ(r) at BCP in the interaction sites of inside complexes are from 0.001 to 0.019 a.u. and from 0.026 to 0.061 a.u., respectively, while these values are in the range of 0.0201–0.0508 a.u. and 0.012–0.098 a.u., respectively, in outside complexes in water and chloroform solvents. The N_34_⋯H_283_ interaction with ∇^2^ρ > 0, H_b_ < 0, 0.5 <  − G_b_/V_b_ < 1 and E_HB_ =  − 55.68 kJmol^−1^ in outside complexes is classified as medium hydrogen bonds. The O⋯H interactions with ∇^2^ρ > 0, H_b_ > 0, − G_b_/V_b_ > 1, and values of energy in the range of − 1.786 to − 55.38 kJ/mol are associated with weak hydrogen bonds. Furthermore, the lower values of topological parameters (ρ and ∇^2^ρ) are observed in the inside complexes, which indicates that outside complexes are more stable than the inside ones. After a detailed examination of the obtained results, we found that the maximum electron density and ${E}_{HB}^{*}$ values belong to the outside complexes in the chloroform solvent, which shows that more strength hydrogen bonds can be observed for the O⋯H and N⋯H interactions. At the same time, the Becke surface analysis is implemented to illustrate the reaction active sites, the strength of the interactions, and to discover the essence of the interactions of DOX with hexakis dimer in a complex. Therefore, the visual molecular dynamics (VMD) program^53^ is used to visualize the point distribution of the Becke surface and also to determine the interaction active sites. The 3‐dimensional BS generated for DOX‐hexakis dimer complexes are shown in Fig. 2.

Close inspection of this figure shows that the three red zones correspond to the high electron density region, which stems from H-bonds. After analysis, numerous surface minima are observed, and at the same time, three surface maxima are found. These surface maxima are meaningful because they have associated with the strength of the hydrogen bond between DOX and hexakis dimer in complexes. The sequence of electron density at these maxima is 0.02 > 0.014 > 0.013, which shows the sequence of H‐bond strength in N–H and O–H interactions. This conclusion is identical to the results of the AIM analysis.

### NCI plot analysis

**Figure 3:**
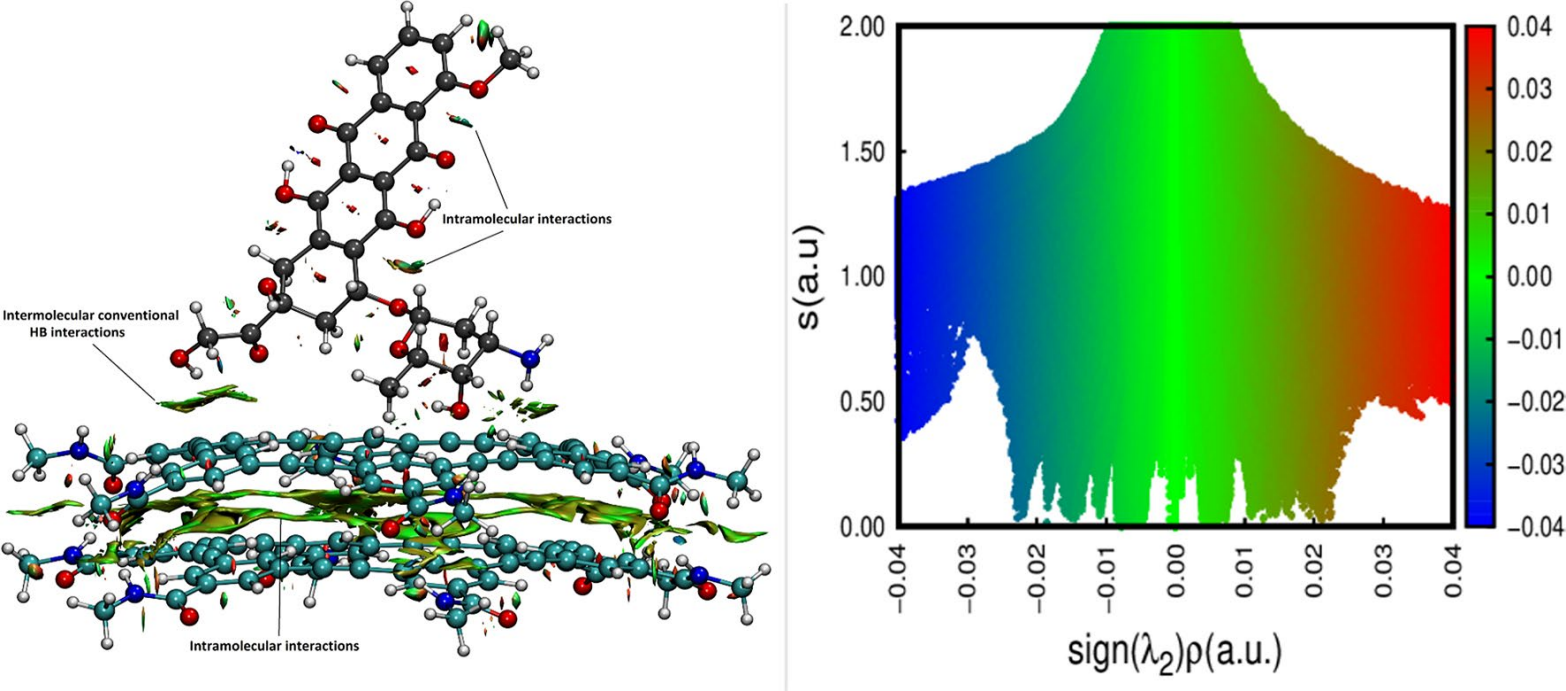
Reduced density gradient isosurface map of DOX/hexakis dimer. The isovalue is set to 0.04 a.u. The value of Sign(λ)ρ in the surfaces is represented by filling color according to the color bar.

The use of NCI index enable the understanding of the interactions in a complex since each method can recognize regions of the weak and strong electron pairing respectively. The NCI index of Yang and co-workers is based on the reduced density gradient (RDG =|∇ρ|/2(3π^2^)^1/3^ρ^4/3^), and also is suitable to define the nature of the weak bonds involved in the structures. The NCI isosurfaces of the reduced electron density gradient between the DOX molecule and hexakis dimer for the most stable complex is depicted in Fig. 3.

The symbol-coding scheme used for isosurfaces extraction are defined as follows: blue indicates the strong attractive interaction, green indicates the intermediate interactions, such as π-π stacking and H-π interactions, while red suggests repulsive interactions. According to the NCI plot (see Fig. 3), the green color that appears around the aromatic rings of the hexakis dimer demonstrates their attractive van der Waals (vdW) interactions. A closer look into the colors in these regions showed that the colors could be mixed and present half red and the other half-blue color. These areas are related to the electrostatic and intramolecular hydrogen bonding interactions between the hydrogen atoms in N–H groups and the oxygen atoms in C=O groups of the two plates of hexakis. It can also be seen from Fig. 3 that the hydrogen bonds appear, blue NCI domains, in the region where the O and N atoms of DOX interact with the hydrogen atoms of the carrier. The attractive vdW interactions are also observed between O atoms of drug and the C atoms of the carrier surface. The result of NCI indicate the hydrogen bonding being responsible for the enhanced binding with the DOX molecule in the DOX-hexakis dimer complexes.

### Electronic structure of DOX and hexakis dimer complexes

Changes in the electronic structure of hexakis dimer upon adsorption of DOX molecule are examined by calculating the energy gaps of frontier molecular orbital and partial density of states. The calculated energy gap (ΔE_LUMO-HOMO_) and the conceptual DFT-based reactivity descriptors are reported in Table 3.

**Table 3:** Calculated energies of frontier molecular orbitals (EHOMO, ELUMO, eV), HOMO–LUMO band gap (Eg, eV), chemical potential (μ, eV), global hardness (ƞ, eV) and global electrophilicity index (ω, eV) of the studied systems.

Model	E_HOMO_	E_LUMO_	Eg	ω	ƞ	μ
Outside complexes	− 7.339	− 2.206	5.132	4.438	2.566	− 4.773
	− 7.215*	− 2.222*	4.993*	4.459*	2.497*	− 4.719*
Inside complexes	− 7.080	− 1.974	5.106	4.014	2.553	− 4.527
	− 6.833*	− 1.926*	4.907*	3.908*	2.454*	− 4.379*

*In water solvent.

As it could be observed in Table 3, (i) the ΔE_LUMO-HOMO_ gap of outside complexes are higher than that of inside complexes. This means that the outside complexes are more stable than the inside complexes. (ii) The negative values of the chemical potential reveal the stability of DOX-hexakis dimer complexes. (iii) While the global hardness (η) is decreased, the electronegativity parameter is increased after the formation of complexes. (iv) The calculated electrophilicity of outside complexes are significantly higher than the value of electrophilicity of inside complexes, especially in chloroform solvent. From these results, we can conclude that these changes could be attributed to the stronger H-bond interactions between DOX and hexakis dimer. To understand the interaction of DOX with hexakis dimer, the electronic structure has been calculated through total density of states and projected density of states, as shown in Fig. S3. As can be seen in this Figure, the TDOS in the most stable complex is strictly the superposition of the PDOS of free DOX drug and hexakis dimer. A comparison of the DOS plot of a free drug molecule with the adsorbed drug at DOX-hexakis dimer complex shows that after adsorption of DOX molecule, DOS of hexakis dimer is further increased and also leads to a significant increase in characteristic features of the DOS plot. These findings show that the electronic properties of complexes do change upon the interaction between DOX and hexakis dimer in drug-carrier complexes.

### GIAO NMR calculations

^1^H NMR chemical shifts (δ) are generally relevant to the detailed understanding of the electronic properties in molecules. In this study, NMR chemical shifts of the amide and aromatic protons of hexakis macrocycles structure are carried out through self‐consistent reaction field (SCRF) theory incorporating the polarization continuum model (PCM). Therefore, to have a comparison between experimental and theoretical chemical shifts, the obtained results are compared with the experimental data. The experimental chemical shift of protons attached to carbon and nitrogen in the aromatic and amide regions of the hexakis monomer is about 7.9–9.08 ppm, which is reported by Gong et al.^12^ and similar to our predicted values (Table S5). Besides, ^1^H NMR calculations are performed on hexakis dimers to understand the dimerization process of (m-PE) macrocycles. The theoretical ^1^H NMR spectrum obtained from amide and aromatic protons of hexakis dimer revealed that these peaks are located at δ = 7.1–8.2 ppm, which indicates reduced molecular motion caused by aggregation. This result is also in good agreement with the ^1^H-NMR data reported by Zhang et al.^13^.

## Conclusions

In the present investigation, the interaction of DOX molecules with hexakis dimer as a novel nanocarrier is studied in chloroform and water solvents via density functional theory calculations, and the following conclusions have been made.

All the ΔE_ad_ values are negative, which indicated that the adsorption of the DOX drug on the hexakis dimer spontaneously proceeded. However, the ΔE_ads_ values for adsorption of DOX drug in the outside of the carrier are greater than those inside it, especially in chloroform solvent. These results can be attributed to the strong hydrogen bond interaction that forms in the outside DOX-hexakis dimer complexes. Regarding the obtained results of EDA, the significant contributions to the total bonding energy are dispersion and electrostatic energies. QTAIM and BS analysis suggested that the existence of hydrogen bonds and non-covalent intermolecular interactions create a reaction of host–guest and keep the stability of complexes. In addition, the calculation results of quantum molecular descriptors revealed that the adsorption of DOX drug on hexakis dimer nanocarrier enhanced the chemical reactivity. Overall, the results obtained from this study provide the nature of the interaction between DOX molecule and hexakis dimer as a novel nanocarrier, which may be useful for making targeted decisions about cancer treatment.

## Supplementary Information

Supplementary Information.

## References

[1] Lotfi M, Morsali A, Bozorgmehr MR (2018). Comprehensive quantum chemical insight into the mechanistic understanding of the surface functionalization of carbon nanotube as a nanocarrier with cladribine anticancer drug. Appl. Surf. Sci..

[2] Charlier J-C (2002). Defects in carbon nanotubes. Acc. Chem. Res..

[3] Zaboli M, Raissi H, Zaboli M (2020). Investigation of nanotubes as the smart carriers for targeted delivery of mercaptopurine anticancer drug. J. Biomol. Struct. Dyn..

[4] Chen Z (2020). Folic acid-modified erythrocyte membrane loading dual drug for targeted and chemo-photothermal synergistic cancer therapy. Mol. Pharm..

[5] Shahabi M, Raissi H (2020). The transport of Idarubicin therapeutic agent using a novel graphene sheet as a drug delivery platform through a biomembrane. J. Mol. Liq..

[6] Pakdel M, Raissi H, Hosseini ST (2020). Evaluation the synergistic antitumor effect of methotrexate–camptothecin codelivery prodrug from self-assembly process to acid-catalyzed both drugs release: A comprehensive theoretical study. J. Comput. Chem..

[7] Haghi A, Raissi H, Hashemzadeh H, Farzad F (2020). Designing a high-performance smart drug delivery system for the synergetic co-absorption of DOX and EGCG on ZIF-8. RSC Adv..

[8] Izadyar M, Khavani M, Housaindokht MR (2015). A combined molecular dynamic and quantum mechanic study of the solvent and guest molecule effect on the stability and length of heterocyclic peptide nanotubes. Phys. Chem. Chem. Phys..

[9] Zhu J (2020). Upconverting nanocarriers enable triggered microtubule inhibition and concurrent ferroptosis induction for selective treatment of triple-negative breast cancer. Nano Lett..

[10] Taherimehr Z, Zaboli M, Torkzadeh-Mahani M (2020). New insight into the molecular mechanism of the trehalose effect on urate oxidase stability. J. Biomol. Struct. Dyn..

[11] Drev M (2020). Self-assembly of multinuclear sandwich silver(I) complexes by cooperation of Hexakis(azaheteroaryl)benzene ligands, argentophilic interactions, and fluoride inclusion. Inorg. Chem..

[12] Jurček O (2020). Heads or Tails? Sandwich-type metallocomplexes of Hexakis (2, 3-di-O-methyl)-α-cyclodextrin. Cryst. Growth Des..

[13] Zhong Y (2016). Hexakis(m-phenylene ethynylene) macrocycles with multiple H-bonding side chains and modified cavities: Altered stacking strength and persistent tubular assembly. Org. Lett..

[14] Zhong Y (2017). Enforced tubular assembly of electronically different Hexakis(m-Phenylene Ethynylene) macrocycles: Persistent columnar stacking driven by multiple hydrogen-bonding interactions. J. Am. Chem. Soc..

[15] Wang Q (2020). Self-Assembly and molecular recognition in water: tubular stacking and guest-templated discrete assembly of water-soluble shape-persistent macrocycles. J. Am. Chem. Soc..

[16] Zhao D, Moore JS (2003). Shape-persistent arylene ethynylene macrocycles: Syntheses and supramolecular chemistry. Chem. Commun..

[17] Pasban S, Raissi H (2021). New insights into Hexakis macrocycles as a novel nano-carrier for highly potent anti-cancer treatment: A new challenge in drug delivery. Colloids Surf. B.

[18] Lu J (2015). Targeted delivery of doxorubicin by folic acid-decorated dual functional nanocarrier. Mol. Pharm..

[19] Wang JX (2015). MicroRNA-532-3p regulates mitochondrial fission through targeting apoptosis repressor with caspase recruitment domain in doxorubicin cardiotoxicity. Cell Death Dis..

[20] Saad SY, Najjar TA, Al-Rikabi AC (2001). The preventive role of deferoxamine against acute doxorubicin-induced cardiac, renal and hepatic toxicity in rats. Pharmacol. Res..

[21] Santos RVT, Batista ML, Caperuto ÉC, Costa Rosa LFBP (2007). Chronic supplementation of creatine and vitamins C and E increases survival and improves biochemical parameters after doxorubicin treatment in rats. Clin. Exp. Pharmacol. Physiol..

[22] Pakdel M, Raissi H, Shahabi M (2020). Predicting doxorubicin drug delivery by single-walled carbon nanotube through cell membrane in the absence and presence of nicotine molecules: A molecular dynamics simulation study. J. Biomol. Struct. Dyn..

[23] Liu H-N (2018). Mitochondrial targeted doxorubicin-triphenylphosphonium delivered by hyaluronic acid modified and pH responsive nanocarriers to breast tumor: In vitro and in vivo studies. Mol. Pharm..

[24] Abazari R (2018). Chitosan immobilization on bio-MOF nanostructures: A biocompatible pH-responsive nanocarrier for doxorubicin release on MCF-7 cell lines of human breast cancer. Inorg. Chem..

[25] Hasanzade Z, Raissi H (2020). Molecular mechanism for the encapsulation of the Doxorubicin in the cucurbit [n] urils cavity and the effects of diameter, protonation on loading and releasing of the anticancer drug: Mixed quantum mechanical/molecular dynamics simulations. Comput. Methods Progr. Biomed..

[26] Gallicchio E, Levy RM (2012). Prediction of SAMPL3 host-guest affinities with the binding energy distribution analysis method (BEDAM). J. Comput. Aided. Mol. Des..

[27] Vasconcelos IB (2017). Host-guest interaction of ZnBDC-MOF+ doxorubicin: A theoretical and experimental study. J. Mol. Struct..

[28] Grimme S, Antony J, Ehrlich S, Krieg H (2010). A consistent and accurate ab initio parametrization of density functional dispersion correction (DFT-D) for the 94 elements H-Pu. J. Chem. Phys..

[29] Zhao Y, Truhlar DG (2008). A prototype for graphene material simulation: Structures and interaction potentials of coronene dimers. J. Phys. Chem. C.

[30] Krishnan R, Binkley JS, Seeger R, Pople JA (1980). Self-consistent molecular orbital methods. XX. A basis set for correlated wave functions. J. Chem. Phys..

[31] Wachters AJH (1970). Gaussian basis set for molecular wavefunctions containing third-row atoms. J. Chem. Phys..

[32] View, G. 3.0, Gaussian Inc., Pittsburg, PA 15106, USA, 2000–2003 Semichem.

[33] Shao Y (2006). Advances in methods and algorithms in a modern quantum chemistry program package. Phys. Chem. Chem. Phys..

[34] Mennucci B (2012). Polarizable continuum model. Wiley Interdiscip. Rev. Comput. Mol. Sci..

[35] Frisch MJ (2003). Gaussian 03.

[36] Baerends, E. J. *et al.**ADF2013, SCM, Theoretical Chemistry, Vrije Universiteit, Amsterdam, The Netherlands*. http://www.scm.com (2014).

[37] Roy RK, Saha S (2010). Studies of regioselectivity of large molecular systems using DFT based reactivity descriptors. Annu. Reports Sect. C.

[38] Geerlings P, De Proft F, Langenaeker W (2003). Conceptual density functional theory. Chem. Rev..

[39] Parr RG, Szentpaly LV, Liu S (1999). Electrophilicity index. J. Am. Chem. Soc..

[40] Lu T, Chen F (2012). Multiwfn: A multifunctional wavefunction analyzer. J. Comput. Chem..

[41] Bader RFW (1990). Atoms in Molecules: A Quantum Theory.

[42] Becke AD (1988). A multicenter numerical integration scheme for polyatomic molecules. J. Chem. Phys..

[43] Contreras-Garcia J (2011). NCIPLOT: A program for plotting noncovalent interaction regions. J. Chem. Theory Comput..

[44] Ditchfield R (1972). Molecular orbital theory of magnetic shielding and magnetic susceptibility. J. Chem. Phys..

[45] Courseille C (1979). Complex daunomycin–butanol. Acta Crystallogr. Sect. B.

[46] Allen FH (1987). Tables of bond lengths determined by X-ray and neutron diffraction. Part 1. Bond lengths in organic compounds. J. Chem. Soc. Perkin Trans..

[47] Taylor R, Kennard O (1983). Comparison of X-ray and neutron diffraction results for the NH⋯ O= C hydrogen bond. Acta Crystallogr. Sect. B Struct. Sci..

[48] Pasban S, Raissi H, Mollania F (2016). Solvent effects on the structural, electronic properties and intramolecular N-HO hydrogen bond strength of 5-aminomethylene-pyrimidine-2, 4, 6 trion with DFT calculations. J. Mol. Liq..

[49] Pasban S, Raissi H, Pakdel M, Farzad F (2019). Enhance the efficiency of 5-fluorouracil targeted delivery by using a prodrug approach as a novel strategy for prolonged circulation time and improved permeation. Int. J. Pharm..

[50] Morokuma K (1971). Molecular orbital studies of hydrogen bonds. III. C= O···H–O hydrogen bond in H2CO···H2O and H2CO···2H2O. J. Chem. Phys..

[51] Nora M (2020). Interactions in inclusion complex of $β$-cyclodextrin/l-Metheonine: DFT computational studies. J. Incl. Phenom. Macrocycl. Chem..

[52] Vatanparast M, Shariatinia Z (2019). Hexagonal boron nitride nanosheet as novel drug delivery system for anticancer drugs: Insights from DFT calculations and molecular dynamics simulations. J. Mol. Graph. Model..

[53] Humphrey W, Dalke A, Schulten KVMD (1996). Visual molecular dynamics. J. Mol. Graph..

