# Supplementary material for: Nanotechnology-based approaches for targeting and delivery of drugs via Hexakis (m-PE) macrocycles
Source: Sci Rep. 2021 Apr 15;11:8256. doi: 10.1038/s41598-021-87011-6 (PMC8050045; doi:10.1038/s41598-021-87011-6)
Supplement: Supplementary file 1 — Supplementary Information. [file 41598_2021_87011_MOESM1_ESM.pdf]

Nanotechnology-Based Approaches for Targeting and Delivery of Drugs via Hexakis (m-PE) Macrocycles

Samaneh Pasban[1] and Heidar Raissi[2]*

[1]Department of Chemistry, University of Birjand, Birjand, Iran

Tel: +985632502064, Email: samaneh_pasban@birjand.ac.ir

[2]Department of Chemistry, University of Birjand, Birjand, Iran

Tel: +985632502064, Email: hraeisi@birjand.ac.ir

* Corresponding E-mail: hraeisi@birjand.ac.ir

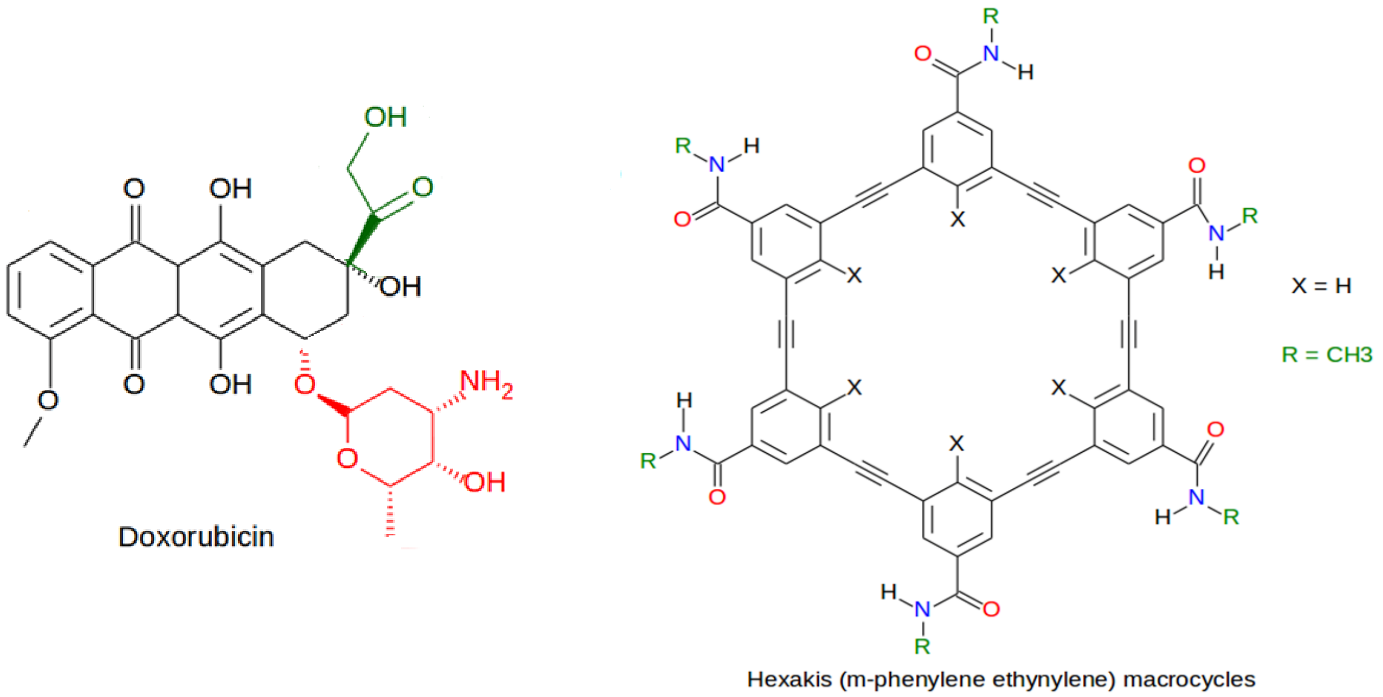

Figure S1 – Molecular structures of doxorubicin (DOX) anticancer drug and hexakis (m-PE) monomer.

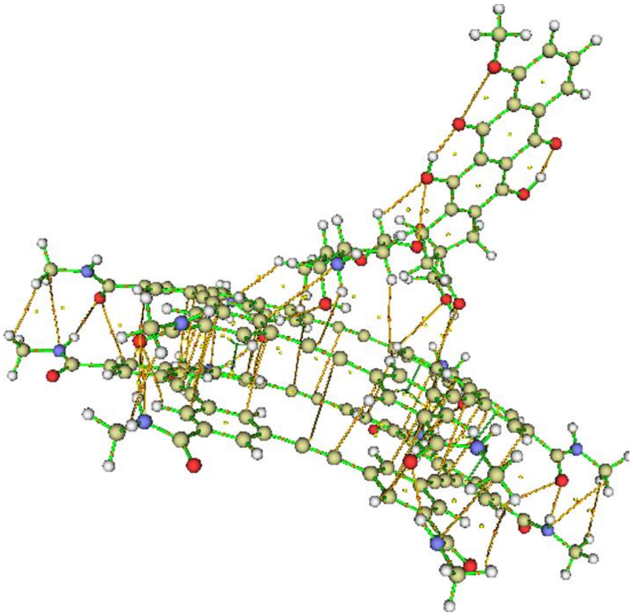

Outside complex

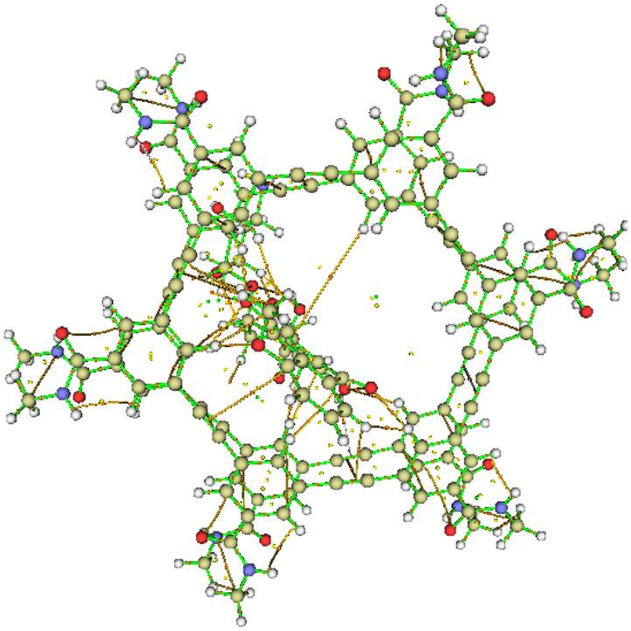

Inside complex

Figure S2 – The molecular graph of the outside and inside complexes obtained from the DFT calculation.

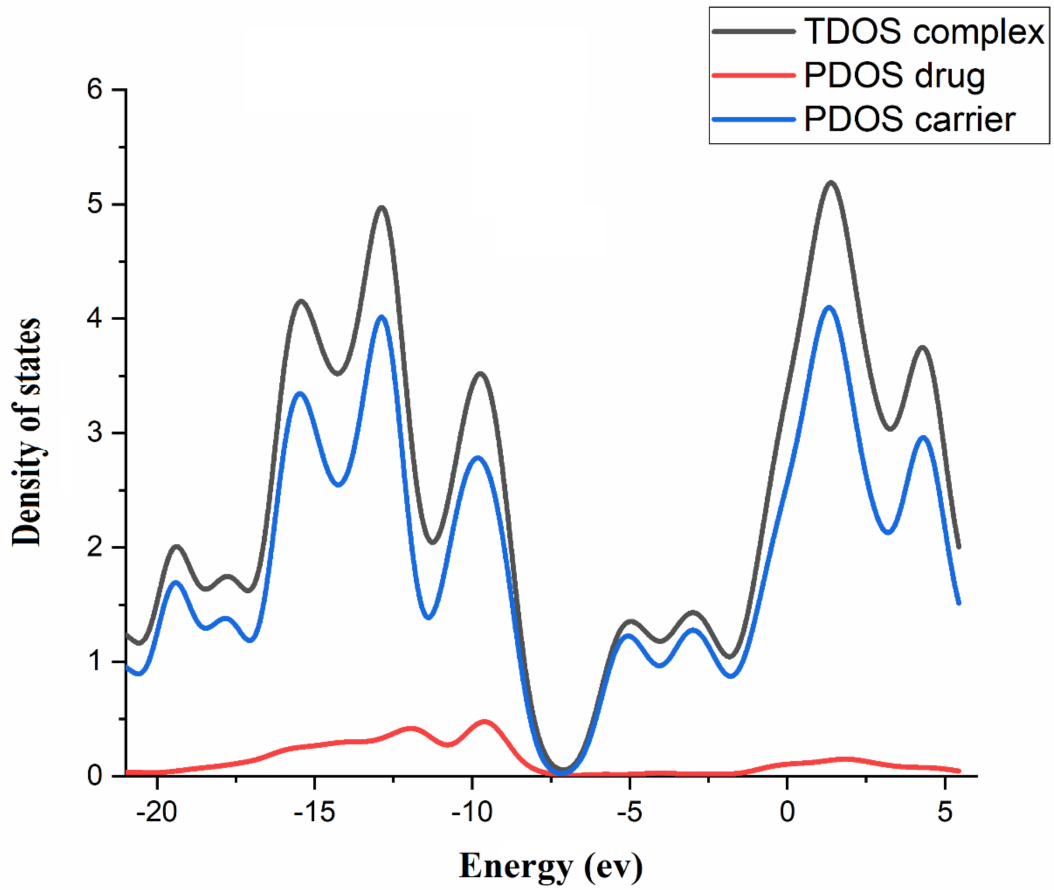

Figure S3 – The calculated electronic density of states for free DOX drug, hexakis dimer carrier, and their complexe.

Table. S1 – The calculated average distance between the N and O atoms and average H-bond length (Å) in water and chloroform for each monomer[a] and hexakis dimer.

Hexakis dimer	***Adsorption site***	***Distance Å***
Chloroform	O115-H109	1.986
Water		2.025
	O132-H105	1.987
		2.007
	O149-H101	1.991
		2.020
	O137-H97	1.988
		2.020
	O127-H125	1.979
		1.999
	O119-H113	1.989
		2.012
	N···O	2.941
		2.947
	N–H	1.338(1.328)
		1.345(1.331)
	C=O	1.012(1.00)
		1.023(1.011)

[a]Values in parentheses refer to calculation for each monomer

Table S2. Computed equilibrium bond lengths (unites in Å) at the binding sites of DOX/hexakis dimer complexes in chloroform and water solvents. Atom labels are defined in Fig.2.

Model	***Adsorption site***	***Distance***
Inside complexes	O_{237}-H_{216}	2.68
		2.72*
	O_{238}-H_{210}	2.21
		2.24*
	O_{234}-H_{196}	2.27
		2.29*
	O_{237}-H_{296}	2.39
		2.42*
	O_{233}-H_{206}	2.28
		2.31*
	O_{229}-H_{288}	2.05
		2.1*
Outside complexes	O_{39}-H_{185}	3.6
		3.91*
	O_{33}-H_{284}	2.2
		2.34*
	O_{37}-H_{277}	2.9
		3.2*
	N_{34}-H_{283}	3.9
		4.2*

* In water solvent.

Table S3. The selected topological parameters of the investigated complexes, the density of the total energy of electrons (H_{BCP}), the kinetic (G_{BCP}) and potential (V_{BCP}) electron energy densities (all in a.u.), $-G_{BCP}/V_{BCP}$ ratio and the hydrogen bond energy (E_{HB}, in kJ/mol) for the intermolecular interactions between DOX drug and hexakis dimer in chloroform and water solution.

	Site adsorption	**ρ_{BCP}**	**$\nabla^2\rho_{BCP}$**	**G_{BCP}**	**V_{BCP}**	**H_{BCP}**	**$-G_{BCP}/V_{BCP}$**	**E_{HB}**
**Inside *complexes***	O_{239}-H_{216}	0.01661	0.0505	0.01160	-0.0105	0.00101	1.0960	-13.901
		0.01658	0.0502	0.01154	-0.0105	0.00100	1.0950	-13.843*
	O_{238}-H_{210}	0.01634	0.0574	0.01267	-0.0109	0.00167	1.1526	-14.431
		0.00817	0.0294	0.00588	-0.0044	0.00146	1.3323	-5.795*
	O_{234}-H_{196}	0.0138	0.0457	0.00996	-0.0085	0.00146	1.1720	-11.158
		0.00649	0.0265	0.00508	-0.0035	0.00153	1.4332	-4.659*
	O_{237}-H_{296}	0.0118	0.0453	0.00935	-0.0073	0.00196	1.2659	-9.7046
		0.00101	0.0156	0.00263	-0.0013	0.00126	1.9326	-1.786*
	O_{233}-H_{206}	0.01307	0.0463	0.0098	-0.0081	0.00171	1.2105	-10.692
		0.00583	0.0202	0.00392	-0.0028	0.00112	1.3999	-3.683*
	O_{229}-H_{288}	0.01939	0.0691	0.01564	-0.0140	0.00163	1.1165	-18.39
		0.01921	0.0614	0.01429	-0.0132	0.00105	1.0796	-17.381*
**Outside *complexes***								
	O_{39}-H_{185}	0.0201	0.0809	0.01786	-0.0155	0.00236	1.1524	-20.347
		0.02239	0.0702	0.01693	-0.0163	0.00061	1.0374	-21.431*
	O_{33}-H_{284}	0.0204	0.0813	0.01803	-0.0157	0.00228	1.1451	-20.677
		0.02142	0.0698	0.0165	-0.0155	0.0009	1.0610	-20.413*
	O_{37}-H_{277}	0.02389	0.0989	0.02231	-0.0199	0.00240	1.1209	-26.134
		0.02251	0.0711	0.01713	-0.0164	0.0006	1.0388	-21.652*
	N_{34}-H_{283}	0.0508	0.01646	0.02315	-0.0421	-0.0190	0.5487	-55.382
		0.0501	0.0121	0.02196	-0.0409	-0.0189	0.5369	-53.685*

* In water solvent.

Table S4. The DOX optimized structural geometries in vacuum phase and experimental data[22]. Lengths are in angstrom (Å)

	EXP[22]	DFT/6-31G**	%SD
C2-C3	1.32[c]	1.35	1.5151
C2-C1	1.39[c]	1.36	-1.1582
C1-C16	1.40[c]	1.39	-0.7142
C15-C16	1.36[c]	1.37	0.7352
C4-C15	1.42[c]	1.40	-1.4084
C3-H3	0.98[c]	1.00	2.0408
C4-C3	1.41[c]	1.40	-0.7092
C2-H2	1.01[c]	1.02	-0.9803
C4-O4	1.34[c]	1.34	0
O4-C23	1.46[c]	1.44	-1.3698
C5-F5	1.34[c]	1.33	-0.7462
C23-H23	1.16[c]	1.13	-2.5862
C5-C15	1.50[c]	1.47	-1.9087
C17-C18	1.37[c]	1.39	0.01459
C18-C12	1.46[c]	1.47	0.6849
C12-C16	1.50[c]	1.49	-0.6666
C12-O12	1.25[c]	1.20	-4.00
C5-O5	1.24[c]	1.24	0
C6-C17	1.40[c]	1.38	-0.7142
C19-C20	1.36[c]	1.37	0.7352
C9-O9	1.44[c]	1.43	-0.6944
O9-H9	1.09[c]	1.05	-3.66
C10-H10	1.01[c]	1.04	2.4798
C13-O13	1.20[c]	1.21	0.8349
C14-H14	0.99[c]	1.01	2.02
C13-C14	1.50[c]	1.49	-0.6666
N22-H22	1.00[c]	0.99	1

c X-ray data (average for four molecules

Table S5. ^{1}H NMR chemical shifts (δ, in ppm) obtained from the DFT calculation, ^{1}H shifts relative to monomer and dimer of hexakis.

Type of H	***Description of Proton***	***Hexakis dimer δ (ppm)***	***Hexakis monomer δ (ppm)***
Ar-H	***aromatic (H is on phenyl ring)***	7.1	7.9
R CO NHR	***amide***	8.2	9.08
R_2N-CH_3	***α to nitrogen (C is attached to N)***	1.2	1.5
